# Technology and Future of Multi-Cancer Early Detection

**DOI:** 10.3390/life14070833

**Published:** 2024-06-29

**Authors:** Danny A. Milner, Jochen K. Lennerz

**Affiliations:** 1 Harvard T. H. Chan School of Public Health, Harvard University, Boston, MA 02115, USA; 2 Union for International Cancer Control, 1202 Geneva, Switzerland; 3 BostonGene, Waltham, MA 02453, USA; joe.lennerz@bostongene.com

**Keywords:** biomarker, early detection, precision oncology

## Abstract

Cancer remains a significant global health challenge due to its high morbidity and mortality rates. Early detection is essential for improving patient outcomes, yet current diagnostic methods lack the sensitivity and specificity needed for identifying early-stage cancers. Here, we explore the potential of multi-omics approaches, which integrate genomic, transcriptomic, proteomic, and metabolomic data, to enhance early cancer detection. We highlight the challenges and benefits of data integration from these diverse sources and discuss successful examples of multi-omics applications in other fields. By leveraging these advanced technologies, multi-omics can significantly improve the sensitivity and specificity of early cancer diagnostics, leading to better patient outcomes and more personalized cancer care. We underscore the transformative potential of multi-omics approaches in revolutionizing early cancer detection and the need for continued research and clinical integration.

## 1. Introduction

Cancer is a complex group of diseases characterized by the uncontrolled growth and spread of abnormal cells within the body. According to global health statistics, cancer remains a leading cause of morbidity and mortality worldwide, with millions of new cases diagnosed each year [1]. Its impact on individuals, families, and healthcare systems is profound.

The objective of this paper is to investigate the potential of multi-omics approaches in revolutionizing early cancer detection. Specifically, we aim to delineate how the integration of diverse data types—genomic, transcriptomic, proteomic, and metabolomic—can overcome the limitations of current diagnostic methods, which often fall short in sensitivity and specificity for early-stage cancers. By providing a comprehensive overview of the existing multi-omics technologies and their applications, we seek to highlight their transformative potential in the realm of oncology. Furthermore, we discuss the inherent challenges in data integration from these heterogeneous sources and present successful case studies from other fields that underscore the utility of a multi-omics strategy. Ultimately, our goal is to underscore the importance of continued research and clinical integration of multi-omics approaches to improve early cancer detection, enhance patient outcomes, and enable more personalized cancer care.

## 2. Early Cancer Detection

Early cancer detection is crucial in mitigating the severity of the disease and improving patient outcomes. At the initial stages, cancer is often asymptomatic or presents with subtle signs, making it challenging to diagnose using traditional methods. As a result, there is a growing imperative to develop advanced diagnostic approaches that enable the identification of cancer at its earliest, most treatable stages, ultimately enhancing the chances of successful intervention and survival.

## 3. Current Challenges in Early Cancer Detection

The current landscape of early cancer detection is marked by significant challenges associated with traditional diagnostic methods. While techniques such as imaging and tissue biopsy have been instrumental in diagnosing various cancers, they often lack the necessary sensitivity and specificity required for detecting cancer at its incipient stages. The limitations of these approaches include their inability to discern subtle molecular changes indicative of early-stage disease and the potential for false positives or negatives. Moreover, many existing diagnostic tools may be invasive, leading to patient discomfort and sometimes discouraging timely screening. Given these limitations, there is a pressing need for the development and implementation of more sensitive and specific approaches that leverage cutting-edge technologies, such as multi-omics, to unravel the molecular intricacies of cancer and enable more accurate and early detection.

**Figure 1:**
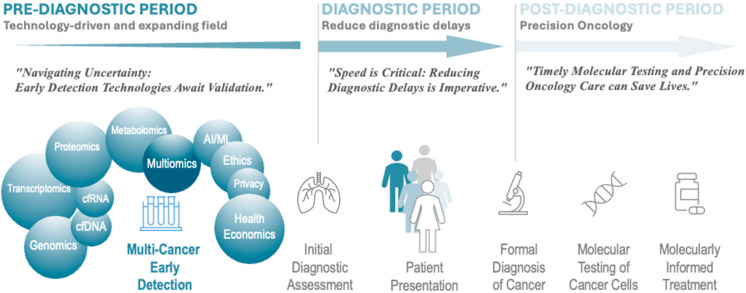
The landscape of multi-omics detection is not only in early detection or pre-diagnosis where there is both an explosion of approaches and technologies but also a whole host of challenges for integration into healthcare for screening or early biomarker detection. The diagnostic period, where a definitive answer must be reached relies on traditional and evolving technologies and establishes the gold standard. For more than two decades, precision oncology in the post-diagnostic period has relied on molecular approaches to select exact courses of therapy, and this approach is currently expanded exponentially across cancer types and medications.

Here, the authors provide an overview of the challenges in the field of Multi-Cancer Early Detection (MCED). Recognizing the limitations of traditional diagnostic methods, and addressing this critical gap, the authors advocate for the exploration and integration of advanced technologies, particularly multi-omics approaches, to unravel the molecular intricacies of cancers (Figure 1). The outline aims to raise awareness of the transformative potential of multi-omics in revolutionizing early cancer diagnostics, urging a collective effort towards the development and adoption of these innovative strategies for improved patient outcomes.

## 4. Multi-Omics Approaches

Multi-omics represents a transformative approach in biology, encompassing a spectrum of disciplines that delve into the intricate molecular landscape of cancer. At its core, multi-omics aims to integrate different dimensions of comprehensive molecular assessments, including genomics, transcriptomics, proteomics, metabolomics, and beyond. This integrative framework empowers researchers to transcend traditional silos, offering a panoramic view of cellular dynamics. By synergistically analyzing diverse molecular layers, datasets grow in size and complexity, and researchers grapple with the daunting task of data integration, interpretation, and standardization. Moreover, ethical considerations necessitate safeguards to uphold data privacy and integrity.

### 4.1. Integration of Multi-Omics Data–Challenges and Benefits of Integrating Data from Different Omics Levels

One of the primary hurdles is the inherent heterogeneity of data generated by different omics technologies. Genomic, transcriptomic, proteomic, and metabolomic datasets often vary in format, quality, and scale, complicating their integration into cohesive analyses [2]. Technical limitations further exacerbate this complexity, as each omics platform possesses distinct sensitivities, dynamic ranges, and biases, necessitating sophisticated computational methods for harmonization and comparison. Moreover, the interpretation of integrated multi-omics data demands a comprehensive understanding of the biological context, considering factors such as temporal dynamics, spatial organization, and regulatory interactions [3]. Despite these challenges, the integration of multi-omics data offers unparalleled insights into biological systems, facilitating a holistic understanding of molecular interactions and emergent properties. By leveraging diverse data sources, researchers can enhance predictive models, unravel complex disease mechanisms, and pave the way for personalized cancer care [4] tailored to individual molecular profiles [5].

### 4.2. Examples of Successful Multi-Omics Integration in Other Fields

In various scientific domains, the successful integration of multi-omics data has revolutionized research paradigms and enabled groundbreaking discoveries. For instance, in cancer research, initiatives like The Cancer Genome Atlas (TCGA) [6] have integrated genomic, transcriptomic, epigenomic, and proteomic data from thousands of patients, unveiling novel cancer subtypes, biomarkers, and therapeutic targets [7]. Similarly, in microbiome studies, projects such as the Human Microbiome Project (HMP) [8] have combined metagenomic, metatranscriptomic, and metabolomic data to elucidate the role of microbial communities in human health and disease. In neuroscience, endeavors like the Neuroimaging and Multi-Omics Study (NIMS) [9] have integrated neuroimaging data with genomic, transcriptomic, proteomic, and metabolomic data to unravel the molecular underpinnings of neurological disorders [10]. Moreover, in agriculture and food science, precision agriculture practices leverage integrated genomic, transcriptomic, metabolomic, and environmental data to optimize crop yields, enhance disease resistance, and improve food quality and safety. These examples underscore the transformative potential of multi-omics integration.

### 4.3. Role of Multi-Omics in Early Cancer Detection—How Multi-Omics Can Enhance Sensitivity and Specificity

The traditional ability to screen for cancer in humans has been limited by very coarse tools (e.g., imaging, symptoms, laboratory tests) that may be expensive, temporally suboptimal, or non-specific. The five most screened for cancers (breast, cervix, colorectal, lung, and prostate) use imaging, invasive procedures, physical exams, and laboratory tests in asymptomatic individuals at a cost ranging from USD 1800 to USD 5500 per patient along with up to a week or more of the patient’s time. Many of these tools have been greatly improved or enhanced (e.g., digital mammography, stool molecular screening, etc.), which have come at an increased cost but with better results. The multi-omics approach can include these five main cancers along with many others depending on the modality and platform. By considering the limit of detection of traditional methods compared with multi-omics in parallel with the breadth of coverage—for tumors for which there is no screening—the overall sensitivity and specificity to detect ANY cancer dramatically increases and at a minute fractional cost per cancer. Individual comparisons of multi-omics versus one screening tool pose challenges for this argument. For example, molecular approaches to breast cancer being suggested as enhancements of the primary tool of mammography are not supported in reverse logically (i.e., screening with molecular tools to follow up with mammography) [11]. Yet, one of the major arguments in the field is the number of false positive multi-omic signatures and the “great cost” to follow up patients with what are possibly inferior tools. Carefully planned and well-designed studies that can address the true sensitivity and specificity impacts of multi-omics are needed at a population level and are forthcoming.

Additional examples of multi-omics enhancing early cancer detection include the identification of circulating tumor DNA (ctDNA) and exosomal RNA in blood samples, which can serve as non-invasive biomarkers for various cancers such as pancreatic, ovarian, and liver cancers. These cancers often lack effective screening methods and are typically diagnosed at advanced stages. By detecting specific genetic mutations, epigenetic modifications, and protein expression patterns associated with these cancers, multi-omics approaches can potentially identify them at much earlier stages. Another example is the use of metabolomics to detect changes in metabolite levels that are indicative of cancer metabolism. For instance, abnormal levels of certain metabolites in urine or blood samples can signal the presence of bladder or kidney cancers, respectively. The integration of proteomics, which analyzes protein alterations, also holds promise for early detection of cancers like gastric and esophageal cancers, where early symptoms are often non-specific. Similarly, the comprehensive characterization of the immune system is a compelling approach to identify and monitor cancer patients [12]. These examples underscore the potential of multi-omics to provide a comprehensive and sensitive approach to cancer screening, enabling earlier intervention and better patient outcomes [13].

### 4.4. Potential for Identifying Early Cancer Biomarkers

Although the impact of improving cancer detection with multi-omics in traditionally screenable cancers remains debatable, there is little argument against using multi-omics for identifying signatures of unscreenable cancers [14] including those that are particularly lethal [15]. When a multi-omics platform is agnostic to tumor type (e.g., sequencing), discovery of new biomarkers is possible though this approach has not been part of the routine commercialized platforms to date. Continued research is needed, including tumor and paired sample sequencing to optimize the earliest and most impactful biomarkers. In existing platforms and approaches, biomarkers for nonscreenable cancers are included which allow, over time, for understanding the impact of these signatures on the stage of detection and outcome. Importantly, when new markers are discovered that indicate earlier stages, they can be added to existing platforms easily.

Furthermore, the integration of advanced machine learning and artificial intelligence (AI) techniques can significantly enhance the identification of early cancer biomarkers from multi-omics data [16]. These technologies can analyze large and complex datasets to uncover patterns and associations that may not be apparent through traditional analytical methods. AI-driven approaches can also improve the sensitivity and specificity of biomarker discovery by reducing the noise and variability inherent in multi-omics data. Additionally, collaborations between academic institutions, research centers, and industry partners are crucial to accelerate the translation of these discoveries into clinical applications. By leveraging the combined expertise and resources of these stakeholders, it is possible to develop robust, scalable, and cost-effective multi-omics platforms that can be integrated into routine clinical practice, thereby improving early detection and patient outcomes for a wide range of cancers.

### 4.5. Technological Advances

#### 4.5.1. Genomic Technologies—Next-Generation Sequencing (NGS) and Its Applications

With nearly two decades of commercial utilization of next-generation sequencing, workflow, efficiency, bioinformatics, and costs continue to improve and expand. Direct tumor tissue sequencing and automated analysis using artificial intelligence can produce a precision profile for an individual patient that dictates therapy, prognosis, and other valuable clinical insights. Such profiling also produces large amounts of data that may or may not have actionable or interpretable information. Similar results and challenges are encountered in focused tumor profiling with NGS (i.e., limited to specific oncological targets). However, NGS now allows for screening patient material (e.g., blood, stool) for the presence of cancer signatures through the examination of hundreds of thousands of pre-specified targets which are analyzed by a bioinformatic approach [17]. Moving this technology into the cancer screening space has been possible because of massive reductions in costs, streamlined analysis, and only actionable results. Unlike traditional tumor sequencing where the pre-analytical sample can be vetted by pathological examination, screening NGS from, for example, peripheral blood may or may not come from a patient with cancer. However, the depth and breadth of sequencing in this approach can identify very small signatures of cell-free DNA and, thus, result in earlier detection.

#### 4.5.2. Single-Cell Genomics for Early Cancer Detection

In parallel with approaches that look for cell-free DNA using NGS, capture and analysis of single-cell genomes through circulating tumor cells can greatly enhance the interpretation of potential cancer signatures [18]. The challenge of this approach is the capture of the cells for which continued development has pointed to several high-quality signatures that are agnostic to tumor type [19]. Once captured, a single cell that represents a malignant population can be analyzed across all oncogenic markers as a system to produce a definitive diagnosis and potential treatment. Moreover, single-cell capture allows for both DNA sequencing and RNA sequencing, providing functional as well as static genetic information [20].

#### 4.5.3. Transcriptomic Technologies in Elucidation and in Screening

Transcriptional analysis of RNA from tumor tissue or peripheral blood has been an invaluable tool for determining the expression states of tumors, leading to unique signatures of cancers. Prognostic or predictive panels from known tumors are now used very commonly in patients with known cancers are guide treatment. Although the very powerful and comprehensive approach of RNA sequencing provides numerous targets for early detection, utilization of these targets in practical assays uses focused detection. A variety of powerful tools and techniques now allow for the examination of RNA-based markers directly from peripheral blood for precision oncology screening [21]. Specific gene expression, microRNA, PIWI-interacting RNA, and small nucleolar RNA, among others, have been identified as valid early biomarkers for breast, colorectal, esophageal, gallbladder, gastric, liver, skin, lung, pancreatic, and kidney cancers [22].

### 4.6. Mass Spectrometry and Its Applications in Cancer Proteomics

Mass spectrometry (MS) is pivotal in cancer proteomics, excelling in identifying, quantifying, and characterizing cancer-related proteins. By ionizing molecules and measuring their abundance based on mass-to-charge ratio, MS enables comprehensive analysis of protein expression, post-translational modifications (PTMs), interactions, and localization. It is instrumental in pinpointing cancer-specific biomarkers by comparing cancerous and normal tissue proteomes, aiding diagnosis, prognosis, and therapy. Additionally, MS elucidates PTMs crucial in cancer signaling. It unveils dysregulated networks and pathways via techniques like affinity purification coupled with MS (AP-MS), fostering the discovery of novel therapeutic targets. In summary, MS is a versatile tool empowering cancer proteomics, driving precision medicine advancements.

### 4.7. Protein Profiling for Early Cancer Detection

Protein profiling, utilizing advanced proteomic technologies, shows promise for early cancer detection [23]. One method involves analyzing circulating proteins in biofluids like blood, urine, or saliva, potentially bearing cancer signatures. Techniques such as liquid chromatography–mass spectrometry (LC-MS) allow high-throughput screening, aiding in the development of minimally invasive diagnostic assays [24]. Additionally, protein profiling on tissue samples from biopsies or surgeries can unveil molecular alterations in early-stage tumors [25]. By comparing cancerous and normal tissue proteomes, specific biomarkers for early cancer stages can be identified, enabling targeted treatments. Integration of multiple biomarkers into panels or signatures enhances diagnostic accuracy, often through machine learning algorithms analyzing complex proteomic datasets. Overall, protein profiling offers promising non-invasive or minimally invasive approaches for early cancer detection, fostering improved patient outcomes through timely intervention [26].

### 4.8. Metabolite Profiling in Cancer Detection

Metabolomic technologies offer a powerful approach to detecting cancer through the analysis of metabolite profiles [27]. In cancer cells, metabolic pathways are often rewired to sustain rapid proliferation and tumor growth, resulting in distinct metabolic signatures that can be detected through metabolomic profiling (e.g., in prostate [28] or breast cancer [29,30]). By quantifying small-molecule metabolites in biological samples such as blood, urine, or tissue, metabolomic techniques such as mass spectrometry and nuclear magnetic resonance spectroscopy can identify aberrant metabolic pathways associated with cancer development and progression. These metabolic alterations can serve as biomarkers for cancer detection, providing valuable insights into the metabolic dysregulation underlying tumorigenesis.

### 4.9. Metabolomics as a Tool for Identifying Metabolic Signatures of Early-Stage Cancer

Metabolomics holds great promise as a tool for identifying metabolic signatures indicative of early-stage cancer. Early-stage tumors often exhibit subtle metabolic changes that precede overt clinical symptoms, making them challenging to detect using conventional diagnostic methods. Metabolomic approaches enable the detection of these early metabolic alterations, offering the potential for early cancer diagnosis and intervention. By analyzing metabolic profiles in biofluids or tissues from individuals at risk of developing cancer, metabolomic technologies can identify specific metabolic biomarkers or signatures associated with the early stages of tumor formation. These metabolic signatures may include alterations in pathways such as glycolysis, amino acid metabolism, or lipid metabolism, providing valuable diagnostic information for early cancer detection and personalized treatment strategies.

A summary of current technologies is provided in Table 1.

## 5. Case Studies and Applications

### 5.1. Successful Examples of Multi-Omics in Early Cancer Detection

One notable example of a successful multi-omics application in early cancer detection is the “CancerSEEK” study [31]. This project, led by researchers at Johns Hopkins University, utilized a multi-omics approach to develop a blood test capable of detecting eight common cancer types at early stages [32]. By integrating genomic and proteomic data, the CancerSEEK test identified circulating tumor DNA mutations and protein biomarkers indicative of cancer presence. Another significant endeavor is the “STRIVE” study (Systematic Tracking of Risk and Early Detection in Gastrointestinal Cancer), which employed multi-omics analyses to identify molecular signatures associated with early-stage gastrointestinal cancers. By integrating genomic, transcriptomic, and proteomic data from tissue samples, STRIVE identified molecular alterations characteristic of early-stage gastrointestinal tumors, enabling the development of targeted screening and diagnostic strategies.

In addition to these prominent studies, the “PanSeer” project represents another successful application of multi-omics in early cancer detection. Researchers from multiple institutions collaborated on this study to develop a blood test capable of detecting five types of cancers up to four years before conventional diagnosis. By analyzing methylation patterns in circulating tumor DNA, the PanSeer test demonstrated high sensitivity and specificity in identifying early-stage cancer, thus offering a promising tool for early intervention.

Furthermore, the “Galleri” test by GRAIL is an exemplary commercial application of multi-omics for cancer detection. The Galleri test leverages next-generation sequencing and machine-learning algorithms to analyze methylation patterns in cell-free DNA. This test can detect more than 50 types of cancer, many of which lack effective early screening methods. Clinical trials have shown that Galleri can identify cancers at early stages with a low rate of false positives, underscoring the potential of multi-omics in revolutionizing cancer diagnostics.

Another noteworthy example is the application of multi-omics in lung cancer screening. The “AURORA” study focuses on integrating genomic, transcriptomic, and proteomic data to identify biomarkers for early lung cancer detection. By analyzing tissue and blood samples from patients at high risk of lung cancer, the AURORA study aims to develop a comprehensive screening tool that can detect lung cancer at its earliest stages, potentially improving patient outcomes significantly.

These successful examples highlight the transformative potential of multi-omics approaches in early cancer detection, offering more comprehensive, sensitive, and specific screening tools compared to traditional methods. As technology advances and more large-scale studies are conducted, the integration of multi-omics into routine clinical practice holds promise for significantly improving early cancer diagnosis and patient prognosis.

### 5.2. Outcomes and Implications

These pioneering studies have yielded promising outcomes and implications for early cancer detection. The CancerSEEK test demonstrated high sensitivity (70–98%) and specificity (99%) in detecting early-stage cancers across multiple types, showcasing the potential of multi-omics approaches for non-invasive cancer screening. The development of multi-omics-based screening tests holds significant implications for clinical practice, offering the possibility of earlier cancer detection, improved patient outcomes, and reduced healthcare costs. Moreover, these studies underscore the transformative potential of multi-omics integration in unraveling the complex molecular landscape of cancer and advancing precision medicine approaches tailored to individual patients. As multi-omics technologies continue to evolve and become more accessible, their widespread adoption in cancer screening and diagnosis holds promise for revolutionizing early detection efforts and ultimately reducing the global burden of cancer.

### 5.3. Challenges and Limitations

There are many challenges and limitations in the utilization of multi-omics approaches to screening which are only somewhat due to the technology itself. Specific to the testing approaches, the limit of detection by stage of cancer is one area in need of ongoing improvement. By increasing data on detection signatures and outcomes, the quality of the testing will be improved and answer many of the pending questions regarding false positives [33], natural history of cancer, and evolution of signature by stage. Another area that is rapidly improving but still in need of resolution with current practice is cost, reimbursement, and timing. The actual individual cost for most of the commercially available tools is less than aggregate-focused screening; however, the total cost impact on health systems for utilizing such tools in place of traditional methods (or in transitions) must be modeled and presented in the best interest of public health and with 100% equitable access [34]. Whether employers, insurance companies, government, or private citizens pay for the screening is highly dependent on the timing of testing and the lifetime testing needed. As epidemiological data grow and improve, an optimal sequencing and timing of testing will emerge but that has not yet been determined. Within the realm of current practice, there remain cultural and personal biases about the technology which stem from a misunderstanding of the term “false positive”, the relative insensitivity of current common tools, and a failure to accept a new paradigm versus rationalization of integration into current systems.

### 5.4. Consider Ethical and Privacy Concerns

Key ethical and privacy concerns related to early cancer detection include the potential misuse of genetic information, such as discrimination in employment or insurance coverage based on predisposition to cancer. Privacy breaches of sensitive health data, especially in the context of genetic testing and biobanking, raise concerns about data security and confidentiality. Moreover, the psychological impact of receiving information about cancer risk or diagnosis, without adequate support and counseling, can lead to anxiety, stress, and stigma. Ensuring informed consent, robust data protection measures and access to supportive resources are imperative to address these ethical challenges and safeguard the rights and well-being of individuals undergoing early cancer detection procedures.

## 6. Future Directions and Implications

### 6.1. Emerging Technologies

The integration of emerging technologies has the potential to significantly impact early cancer detection by improving the sensitivity, specificity, and accessibility of screening methods. Liquid biopsy, for instance, allows for minimally invasive and repetitive sampling, enabling real-time monitoring of tumor dynamics and treatment response. Single-cell sequencing provides insights into intratumoral heterogeneity and clonal evolution, guiding personalized treatment decisions and early intervention strategies [35,36]. Advanced imaging techniques offer high-resolution visualization of tumors and metastases [37], enabling early detection and precise localization of cancer lesions. Moreover, AI-driven analysis of multi-omics data sets enhances the identification of biomarkers and predictive signatures for early cancer detection, facilitating the development of non-invasive and cost-effective screening tests [38,39]. The combination and clinically meaningful integration of these solutions is considered one approach to overcoming the challenges related to early cancer detection [40].

### 6.2. Integration with Clinical Practice: How Can Multi-Omics Approaches Be Incorporated into Routine Clinical Care?

Today, with available commercial tests, patients or systems of patients may present to their primary care doctor or directly to an oncologist with a positive screening result. In a patient with a history of cancer with a similar signature, follow-up is straightforward. In a new patient with no history of cancer—especially young, healthy, non-smokers—who presents with a positive test, there are several options. If the cancer has available work-up modalities (e.g., colonoscopy, prostate ultrasound, etc.), true positives and false positives can be evaluated with the latter being controversial (i.e., when the multi-omics test is more sensitive than the follow-up). If the cancer does not have an available work-up, PET-CT may be the only next step to evaluate a patient. Clinical trials of patients—especially young, healthy, non-smokers—with low-dose pan-tumor regiments like PD-L1 checkpoint inhibitors paired with imaging, may be extremely valuable for future use.

To think of the future of multi-omics for cancer screening and early detection, we must start with a clean slate and work backward. Assume a system has no screening available of any kind. This model is directly applicable to most low- and low-middle-income countries. Based on our population distribution, cancer epidemiology, and age of presentations, we can easily calculate a matrix of detection points for male and female patients across a lifetime which optimizes detection for the known cancers. We now need to include follow-up protocols for positive signatures, which could be as simple as a pan-tumor medication regimen for a specific period, followed by a repeat assay. This model is particularly valuable (as is the multi-omics test) if a signature is included in the detection for the burden of tumor (i.e., transcriptional, metastatic, size-related impacts) because it would stratify patients into those which were likely radiologically detectable and those that were not [41]. The former can be followed with PET-CT to determine the total tumor burden and the latter could have a treat and repeat protocol. In this model, many current tools, services, and systems are either not needed or greatly reduced in need systematically. However, overall, the system is logarithmically less expensive, more efficient, and more amenable to equitable outcomes. But the current systems in high-income countries do not start with a clean slate. There are large economies associated with screening processes including millions of jobs, logistic supply chains, professional services, etc., into which yet another screening modality is trying to be included. Embracing the creative destruction of the disruptive innovation that is a functioning early detection system is the inevitable conclusion where political and financial influences are removed. However, stakeholders will fight against such an approach even when it is ethically and morally no longer legitimate to do so.

### 6.3. Challenges and Opportunities for Translation from Research to Clinical Application

Moving new biomarkers discovered in laboratories to the clinical phase can happen more aggressively when the platforms used for research are mirrored by clinical test platforms. When research approaches are new, untested methods, moving to market may take much longer. Fortunately, the technologies that are currently leading the way in commercial multi-omics approaches are based largely on techniques with more than a decade of clinical use. New discoveries of more powerful biomarkers—especially in populations that have not yet been subjected to high degrees of sequencing scrutiny—will be more quickly adopted as they are simply additions to an existing array.

## 7. Conclusions

Emerging technologies offer transformative potential for early cancer detection, including liquid biopsy, single-cell sequencing, advanced imaging, and AI-driven analytics, enhancing treatment efficacy and patient outcomes or surrogate endpoints [42]. Multi-omics integration is pivotal, combining genomic, transcriptomic, proteomic, and metabolomic data to understand cancer biology, discover biomarkers, and tailor personalized screening and diagnostics. Continued research collaboration and investment are crucial to translating these technologies into clinical practice, driving innovation, and ensuring equitable access. Prioritizing research and development in early cancer detection will advance science and medicine, benefiting cancer patients globally.

## Figures and Tables

**Table 1:** Summarizing the key technologies used in multi-omics approaches for early cancer detection.

Technology	Examples	Promises	Challenges
Genomic Technologies	Next-generation sequencing (NGS), Single-cell genomics	High sensitivity, precision profiling, early detection	High cost, data interpretation complexity, potential for false positives
Transcriptomic Technologies	RNA sequencing, gene expression analysis	Identifies unique cancer signatures, aids in treatment decisions	Data integration complexity, interpretation of results, need for sophisticated bioinformatics tools
Proteomic Technologies	Mass spectrometry (MS), liquid chromatography–mass spectrometry (LC-MS), protein profiling	Identifies cancer-specific biomarkers, minimally invasive, high throughput	Variability in protein expression, technical challenges in quantification, integration with other omics data
Metabolomic Technologies	Mass spectrometry, nuclear magnetic resonance (NMR) spectroscopy	Detects metabolic changes, potential for early diagnosis	Complexity in data analysis, need for large and diverse datasets, standardization of methods
Multi-omics Integration	CancerSEEK, The Cancer Genome Atlas (TCGA), Human Microbiome Project (HMP)	Comprehensive molecular insights, enhanced diagnostic accuracy, personalized medicine	Data heterogeneity, technical limitations, computational challenges, ethical and privacy concerns
Liquid Biopsy	Circulating tumor DNA (ctDNA) analysis, circulating tumor cells (CTCs)	Minimally invasive, real-time monitoring	Sensitivity and specificity issues, need for validation and standardization
AI-driven Analytics	Machine learning algorithms for biomarker discovery and predictive modeling	Enhanced data analysis, identification of complex patterns	High computational demand, data privacy issues, need for robust validation
Advanced Imaging Techniques	High-resolution MRI, PET-CT, optical imaging	Precise localization, improved visualization of tumors	High cost, potential for false positives, need for integration with molecular data
